# Traumatic brain injury causes selective, CD74-dependent peripheral lymphocyte activation that exacerbates neurodegeneration

**DOI:** 10.1186/s40478-014-0143-5

**Published:** 2014-10-20

**Authors:** Richard P Tobin, Sanjib Mukherjee, Jessica M Kain, Susannah K Rogers, Stephanie K Henderson, Heather L Motal, M Karen Newell Rogers, Lee A Shapiro

**Affiliations:** 1 Department of Surgery, Texas A&M University Health Science Center, Temple, TX USA; 2 Central Texas Veterans Health Care System, Temple, TX USA; 3 Department of Anthropology, University of Texas, Austin, TX USA; 4 Department of Surgery, Baylor Scott and White Health, Temple, TX USA; 5 Texas A&M Health Science Center, Baylor Scott and White Health, 702 SW. HK Dodgen Loop, MRB. Rm. 114B, Temple, 76504 TX USA

**Keywords:** TBI, FPI, Fluid percussion injury, CD74, Neuroinflammation

## Abstract

**Introduction:**

Traumatic brain injury (TBI), a significant cause of death and disability, causes, as in any injury, an acute, innate immune response. A key component in the transition between innate and adaptive immunity is the processing and presentation of antigen by professional antigen presenting cells (APCs). Whether an adaptive immune response to brain injury is beneficial or detrimental is not known. Current efforts to understand the contribution of the immune system after TBI have focused on neuroinflammation and brain-infiltrating immune cells. Here, we characterize and target TBI-induced expansion of peripheral immune cells that may act as potential APCs. Because MHC Class II-associated invariant peptide (CLIP) is important for antigen processing and presentation, we engineered a competitive antagonist (CAP) for CLIP, and tested the hypothesis that peptide competition could reverse or prevent neurodegeneration after TBI.

**Results:**

We show that after fluid percussion injury (FPI), peripheral splenic lymphocytes, including CD4+ and CD8+ T cells, regulatory T cells (Tregs), and γδ T cells, are increased in number within 24 hours after FPI. These increases were reversed by CAP treatment and this antagonism of CLIP also reduced neuroinflammation and neurodegeneration after TBI. Using a mouse deficient for the precursor of CLIP, CD74, we observed decreased peripheral lymphocyte activation, decreased neurodegeneration, and a significantly smaller lesion size following TBI.

**Conclusion:**

Taken together, the data support the hypothesis that neurodegeneration following TBI is dependent upon antigen processing and presentation that requires CD74.

**Electronic supplementary material:**

The online version of this article (doi:10.1186/s40478-014-0143-5) contains supplementary material, which is available to authorized users.

## Introduction

Traumatic brain injury (TBI) is a leading cause of death and disability in the United States and throughout the world [1]-[3]. To date, there are no effective treatments to prevent or reverse damage after TBI. Due to the direct impact on the brain, efforts at diagnosis and treatment of TBI have been primarily focused on the central nervous system (CNS). However, it is now well established that peripheral immune mechanisms are involved in the pathology associated with TBI [4]-[7]. The paradigm that has been widely accepted is that the blood brain barrier (BBB) serves as the primary obstacle to the recruitment of leukocytes that could otherwise cause harm to the brain [7],[8]. However, after TBI, there is BBB breakdown and one result is the passage of peripheral immune cells into the CNS [7].

In general, an immune response will begin with an innate and immediate response to injury that includes activation and migration of neutrophils, dendritic cells, macrophages (including microglial cells in the CNS), as well as other less defined cellular and molecular components [9]-[11]. The innate immune response is typically followed by the antigen-specific, adaptive immune response. The transition from an innate to an adaptive response is dependent upon successful antigen processing and presentation, by professional antigen presenting cells (APCs), to antigen-specific T lymphocytes [12],[13]. How, or if, damage to the brain, often described as an “immune-privileged” site [14], elicits an innate, and the transition to, an adaptive immune response from peripheral immune mediators to the CNS, is not known. Evidence for the involvement of antigen processing and presentation in the CNS is provided by the study of Slavin et al. [15]. This study demonstrated that mice lacking the machinery for processing and presentation of full-length myelin basic protein did not develop experimental autoimmune encephalomyelitis (EAE), a mouse model for Multiple Sclerosis (MS). However, when these same mice were exposed to peptides derived from full-length myelin basic protein, the mice became susceptible to EAE. Interestingly, Ling et al. demonstrated that TBI results in recruitment of antigen-specific CD8+ T cells, only in the presence of the antigen for which they are specific [16]. Furthermore, in a model of optic neuritis, the acute immune response leads to an adaptive, specific, anti-brain response [17]-[19]. Conflicting reports suggest that this effect could be either harmful or helpful to recovery [5],[6]. Regardless of their role in the outcome, these findings provide evidence for an adaptive immune response in the brain after TBI.

Efficient antigen processing and presentation via the major histocompatibility complex II (MHCII) pathway in APCs, including macrophages, microglia, dendritic cells, B cells, and γδ T cells, facilitates the transition between innate and adaptive immunity [13],[20]-[22]. Antigen processing and presentation by these professional APCs involves invariant chain (CD74). CD74 serves as a chaperone for MHCII until CD74 is proteolytically cleaved into Class II invariant peptides (CLIP) that fill the antigen-binding groove of MHCII molecules [12],[23]. Replacement of CLIP by antigenic peptides and T cell recognition of antigenic peptide in MHCII is the first step in T cell activation, and the first event in a T cell-mediated adaptive immune response [13],[20],[24]. This vital step of peptide exchange between innate and adaptive immunity has not been elucidated following TBI. Critical to this process is the cleavage of full-length CD74 into CLIP, the loading of CLIP into the antigenic binding groove of MHCII, followed by the replacement of CLIP with antigenic peptides [23]. Therefore, we engineered and synthesized a peptide that competitively antagonizes the antigen-binding site of MHCII, to determine if antagonism of CLIP/antigenic peptide exchange might prevent or reverse the detrimental effects of TBI [25]. Thus, this study was designed to determine if exchanging CLIP for antigenic peptide is important in secondary neurodegeneration following TBI.

## Materials and methods

### Treatment with competitive antagonist peptide (CAP)

CAP was synthesized as previously described [25]. Briefly, using computational design, we identified a 9mer peptide, with 8 total amino acid flanking regions, that by, peptide binding analysis software (MHCPred and netMHC), was predicted to have a higher binding constant than the invariant peptide CLIP for the peptide-binding groove of known MHCII alleles. CAP was synthesized by Elim Biopharmaceuticals. The mice were injected intraperitoneally (i.p.) (1 mg/kg) with CAP. CAP was initially dissolved at 5 mg/mL in dimethyl sulfoxide (DMSO), after which 5 μl of CAP dissolved in DMSO was further diluted with 195 μl of sterile saline and injected (I.P.). In vehicle injected mice, an injection containing 5 μl DMSO dissolved in 195 μl of sterile saline.

### Animals

6-week-old male C57BL/6 J (N = 16) were purchased from Jackson Labs. Invariant chain deficient (CD74^Def^) mice (C57BL/6 background; N = 16) were purchased from Jackson Labs and bred at the Scott and White Healthcare animal facility to maintain homozygous CD74 deficiency. Because littermates are not available to compare with the CD74^Def^ mice (as they are bred to homozygosity), we have used age and sex matched C57BL/6 mice (the background strain for the mice). All mouse care, housing, and experimental procedures were approved by the Baylor Scott and White Healthcare Institutional Animal Care and Use Committee, reference number 2013-021. Wild type mice were divided into four groups for flow cytometry and multiplexing and sacrificed at 24 hrs after TBI. These four groups were: sham + vehicle (N = 6); sham + CAP (N = 5); FPI + vehicle (N = 7 WT); FPI + CAP (N = 6). For the CD74^Def^ mice, two groups were used. These two groups were: Sham (N = 7 CD74^Def^) and FPI (N = 6 CD74^Def^). In addition, we used separate mice for histological studies and these mice were sacrificed 3 days after TBI (N = 6 WT sham + vehicle, 5 FPI + CAP, and 5 CD74^Def^). The three day post-TBI time point was selected because our previous study indicated that this is when the peak of TBI-induced neurodegeneration was observed [26].

### Fluid percussion injury (FPI)

FPI was induced as previously described [26]. Briefly, mice were anesthetized with isoflurane, prepped, cleaned, and shaved, then put into a stereotactic instrument (Stoelting, Inc.). A 2 mm craniotomy was made over the left parietal cortex making sure to keep the dura intact. The female end of a luer lock syringe was cemented over the craniotomy and attached to the fluid percussion apparatus. A 12 16 ms FPI was delivered at a pressure of ~1.4-1.6 atm. Sham mice received identical treatment except the pressure pulse was never delivered. After injury or sham, suture was used to close the scalp over the wound and mice were returned to their home cage resting on a heating pad. Mice were monitored to ensure that they resumed walking, feeding, drinking and grooming behavior.

### Flow cytometry

Mice were sacrificed and spleens were removed. The tissues were dissociated, single cell suspensions prepared, and red blood cells were lysed using GEY'S buffer [27]. The dissociated splenocytes were treated with FC Block (BD Bioscience) and then stained with the following antibodies; CD3, CD4, CD8, CD25, MHCII (clone M5/114.15.1), B220, γδ-TCR (BD Bioscience), and CLIP (15G4, Santa Cruz Biotechnology). For regulatory T cell (Treg) staining we used the eBioscience mouse regulatory T cell staining Kit according to the manufacturer's directions. Live cells were assessed using the Life Technologies LIVE/DEAD® Fixable Aqua Dead Cell Stain Kit according to the manufacturer's directions. Splenocytes were analyzed using a BD FACS Canto II flow cytometer and data were analyzed using FlowJo software (TreeStar Inc.). See Additional file 1: Figure S1 for gating strategy.

### FluoroJade C method

Fluorojade C staining took place as previously described [26]. Briefly, every 6th 50 um section was analyzed within the margins of the lesion. This corresponded to 5-7 sections per mouse. Sections were mounted onto gelatin-coated slides and FluoroJade C histology was performed as previously described [26]. The slides were allowed to air dry, after which cover slips were applied using Vectashield with DAPI (Vector Labs). Prior to analysis, slides were coded to ensure that the reviewer was blind to the condition of the mice. Sections were then visualized using an Olympus IX81 (Olympus Inc., Tokyo, Japan) inverted microscope equipped to visualize FITC. We used the following Olympus UplanS objectives to capture the images: 10× NA0.40 ∞/0.17 FN26.5; 20× nA0.75 ∞/0.17 FN26.5; 40× NA0.75 ∞/0.17 FN26.5. Images were captured using the FV1000 and the Fluoview software (V. 1.7.1.0). Images were saved as 24 Bit Tiff files and brightness and contrast were adjusted using Adobe Photoshop (V. 12.0). For quantitative analysis of Fluorojade C labeling, the peri-lesion area was traced and 400 μm^2^ grids were randomly placed throughout the traced area. All cells which fell within the grid, or contacted the left and upper borders of the grid were included in the counts, whereas those that touched the right or lower border were excluded. The peri-lesion area consisted of +/- 1 mm anterior/posterior (AP) from the injury focus (AP +1.5 mm; medio-lateral: 1.2 mm).

### Measurement of lesion size

Superficial lesion size was measured using a digital caliper (Mitutoyo 150 mm, .01 mm accuracy) as previously described [28],[29]. In order to confirm superficial lesion measurements made with the digital caliper, we assessed the margins of the lesion in the anterior posterior plane, and calculated the expanse of the lesion using a mouse atlas [30]. Initially, every 6th 50 μm Nissl stained section was used for determination. However, in some cases, it was necessary to examine serial sections in which Fluorojade C (or other stains not reported in this manuscript) was stained, in order to confirm that we had accurately accounted for the most anterior and posterior margins of the lesion. It should be noted that considering the margin of error of the digital caliper (100 μm) and the distance between brain slices in the mouse atlas (100 140 μm), all measurements were within the margin of error of these 2 sources.

### Cytokine analysis

Fresh, non-perfused tissue was isolated from the ipsilateral cortex including the lesion area and flash frozen in liquid nitrogen. Frozen tissue was homogenized following the manufacturer’s instructions (Milliplex MAP kit, Millipore). Protein was estimated with a Bradford assay and similar concentrations were made such that 25 μl of homogenate was added to 25 μl of assay buffer. Then, 25 μl of magnetic beads coated with specific antibodies (MCYTOMAG-70 K-PMX, Milliplex MAP Kit, Millipore) was added to this solution and the reaction was incubated at 4 C for 24 hours. Next, the beads were washed and incubated with 25 μl of biotinylated detection antibody at room temperature (RT) for 2 hours. Finally, 25 μl of Streptavidin–Phycoerythrin conjugate compound was added and incubated for 30 min at RT. The beads were then washed and incubated with 150 μl of sheath fluid for 5 min at RT. The concentration of the analytes is then determined by Bio-Plex Manager software version 5.0. The assays were run in triplicate to confirm the results. Analytes were normalized to total protein concentration. In cases where the cytokines in the brain tissue of three or more mice for a group were below the detection threshold, the data were not presented in the graphs.

### Statistical analysis

Statistical analysis was performed using GraphPad Prism 6 (GraphPad Software Inc.). For comparisons between splenocytes from C57BL/6 J and CD74^Def^f, a *t*-test was used with a significance cut-off of P <0.05. For all other analysis, repeated measures ANOVA was used with post-hoc planned comparisons using Dunnett’s correction factor.

## Results

### Expansion of peripheral lymphocytes following Fluid Percussion Injury (FPI)

**Figure 1:**
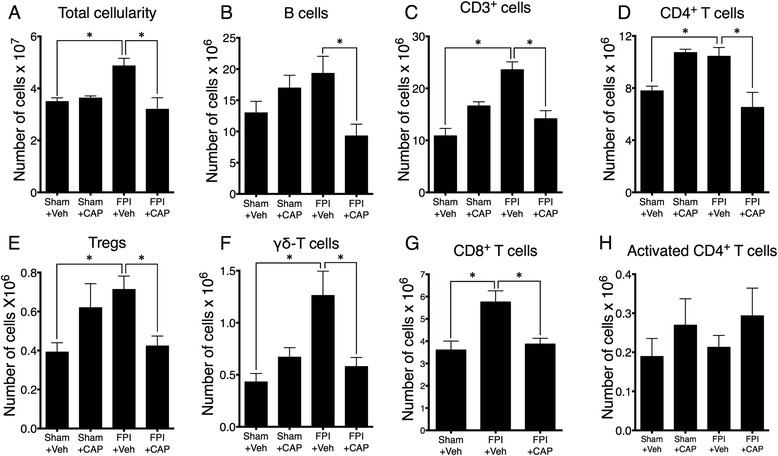
**Selective expansion of splenic immune cell subsets in response to FPI. (A)** The number of viable cells in the spleens (cellularity) of C57BL/6 mice 24 hours following sham surgery or FPI. TBI significantly increased the number of viable cells. **(B)** The number of B cells (B220^+^) showing a trend toward B cell expansion after FPI that does not reach significance. However, treatment with CAP significantly reduced B cells after FPI. **(C)** Analysis of T cells (CD3^+^) revealed a significant expansion after FPI that is significantly reduced following CAP treatment. **(D)** A subset of CD3+ T cells, CD4^+^ T cells (CD3^+^CD4^+^), are also significantly elevated after FPI. This elevation is significantly reduced following CAP treatment. **(E)** Identification of Tregs (CD3^+^CD4^+^CD25^Hi^FoxP3^+^) also revealed a significant increase in this cellular population after TBI. Similar to other T cell populations, this population is also significantly decreased after CAP treatment. **(F)** A similar pattern of FPI-induced increase is observed for γδ T cells (CD3^+^γδ-TCR^+^) and this increase is significantly reduced by our CAP treatment. **(G)** Analysis of CD8^+^ cytotoxic T lymphocytes (CD3^+^CD8^+^) also revealed a significant increase after FPI that is significantly reduced with our CAP treatment. **(H)** Alternatively, no significant differences were observed for activated CD4^+^ T cells (CD3^+^CD4^+^CD25^+^FoxP3^−^) in the spleens of C57BL/6 mice 24 hours following sham surgery or FPI. Sham + vehicle (N = 6); sham + CAP (N = 5); FPI + vehicle (N = 7 WT); FPI + CAP (N = 6). The symbol * indicates P < 0.05.

To characterize the changes in peripheral immune cells following TBI, splenic lymphocytes were harvested at 24 hours after FPI and analyzed using flow cytometry. The results showed that the overall splenic cellularity was significantly increased (P < 0.017) in FPI-treated mice compared to sham-treated controls and this effect was prevented by administration of competitive antagonist peptide (CAP) (Figure 1A). While there was a trend toward increased B220+ B cells (Figure 1B), there was a significant (P < 0.03) reduction in B cell numbers following CAP treatment after FPI (Figure 1B). In addition, there was a significant (P < 0.001) FPI-induced increase in the total number of CD3+ T cells that was significantly (P < 0.001) reduced by CAP treatment after FPI (Figure 1C). Specifically, CD4+ T cells were also significantly increased in number (P < 0.014) and their numbers were significantly reduced (P < 0.018) following CAP treatment (Figure 1D). There were also significant increases in Tregs (P < 0.018), as defined by CD3+, CD4+, high CD25 and expression of the transcription factor FoxP3 (Figure 1E), γδ T cells (P < 0.0.14); (Figure 1F) and CD8+ T cells (P < 0.02); (Figure 1G), all of which were significantly reduced by treatment with CAP (Figures 1E-G). In contrast, there was no significant increase in the numbers of conventional activated CD4+ T cells, nor was there a significant reduction of these cells following CAP treatment (Figure 1H).

### FPI-induced changes in components of antigen presentation by peripheral lymphocytes

**Figure 2:**
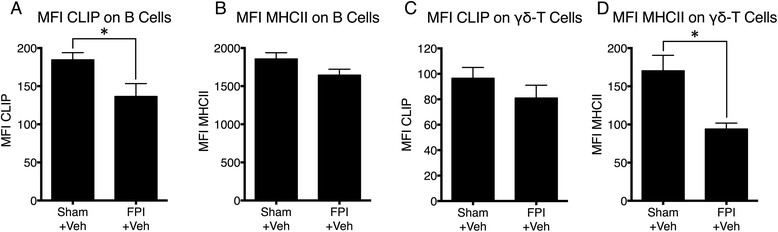
**Differential expression of MHCII and CLIP on B and γδ T cells in response to FPI. (A)** The mean fluorescence intensity (MFI), indicative of the level of cell surface expression of CLIP is significantly reduced on the surface of B cells at 24 hours after FPI. This reduction in CLIP likely reflects replacement of CLIP by other peptides. **(B)** Consistent with this interpretation, no corresponding significant decrease in the MFI of MHCII on the surface of B cells was observed at 24 hours after FPI. **(C)** In contrast, the MFI of CLIP on the surface of γδ T cells remained unchanged, **(D)** while the level of MHCII significantly decreased at 24 hours after FPI. Taken together, these data suggest that B cells are a likely candidate as APCs after FPI. Sham + vehicle (N = 6); FPI + vehicle (N = 7). The symbol * indicates P < 0.05.

A hallmark of the transition between innate and adaptive (specific) immunity is successful antigen processing and presentation. Antigen processing requires proteolytic cleavage of invariant chain (CD74) into peptides known as class II invariant peptide (CLIP). CLIP functions as a “placeholder” for antigen specific binding to the peptide-binding groove of MHCII molecules. B cells and γδ T cells are among cells that are well characterized as professional APCs [31],[22]. Therefore, we examined the effects of FPI on CLIP and MHCII from peripheral lymphocytes. We observed a significant decrease in cell surface CLIP on B cells (Figure 2A). Conversely, we observed no corresponding changes in the cell surface levels of MHC II on B cells (Figure 2B). In contrast to CLIP on B cells, we observed no significant differences in the level of CLIP per γδ T cells (Figure 2C), but a significant decrease in MHCII on γδ T cells (Figure 2D). These results suggest possible FPI-induced alterations to molecular machinery involved in antigen processing and presentation.

To test the effect of our peptide on influencing the transition between innate and adaptive immunity, we examined CLIP and MHCII expression on B cells and γδ T cells in mice administered CAP 20 minutes after FPI. We examined these two cell types because they are known to express MHCII and present antigens. We observed no significant differences in CLIP expression on the cell surface of B220+ B cells (Additional file 2: Figure S2) following CAP administration after FPI. These results suggest that of these 2 potential APCs (B cells or γδ T cells), the data are more consistent with B cells functioning as APCs in response to TBI. In contrast, the data are less suggestive of γδ T cells serving as APCs based on reduced MHCII (Figure 2D) and therefore a higher ratio of CLIP to MHCII on γδ T cells.

### Absence of FPI-induced lymphocyte expansion in invariant chain deficient mice

**Figure 3:**
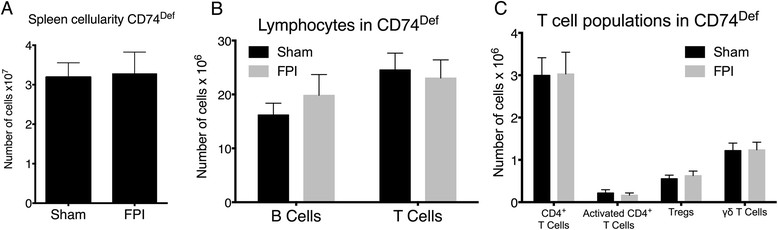
**FPI-induced alterations in peripheral lymphocytes require CD74. (A)** The number of viable cells in the spleens of CD74^Def^ mice is unchanged at 24 hours following sham surgery or FPI. **(B)** Similarly, the number of B Cells (B220^+^) and T cells (CD3^+^) in the spleens of CD74^Def^ mice are not significantly different at 24 hours after sham surgery or FPI. **(C)** Analysis of specific populations of T cells also revelaed no significant differences at 24 hours after sham surgery or FPI. For these experiments we used 5 sham surgery mice and 6 FPI treated mice. The symbol * indicates P < 0.05.

Because we observed changes in CLIP expression resulting from FPI, we sought to determine the significance of CLIP involvement in immune modulation by FPI, by using mice deficient in invariant chain, CD74. CD74, which is required for processing and presentation of full-length proteins [18],[15], can serve as a survival factor and can function as a growth promoting receptor. In support of the notion that CD74 is required for lymphocyte expansion following FPI, mice lacking CD74 exhibited no net change in spleen cellularity 24 hours following FPI (Figure 3A). Alternatively, wild type mice exhibit a significant expansion of lymphocytes in the spleen at 24 hrs after FPI (Figure 1A). Furthermore, there were no changes in numbers or activation state of any of the subsets of B or T cells (Figure 3B and C, respectively).

### CLIP antagonism or invariant chain deficiency modifies the cytokine profile in the CNS following FPI

**Figure 4:**
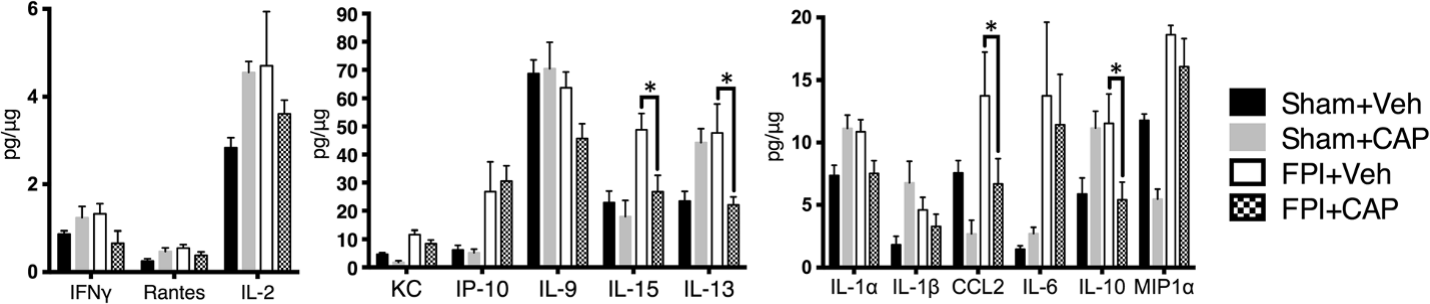
**CAP decreases expression of pro-inflammatory cytokines in the brain following FPI.** Multiplex analysis of cytokines in tissue from the ipsilateral cortex of C57BL/6 mice following FPI. Note the significant reductions in IL-13, IL-15, CCL2 (MCP1), and IL-10. The reduction of this subset of chemokines and cytokines is consistent with the reduction in cells capable of producing these inflammatory cytokines. Sham + vehicle (N = 4); sham + CAP (N = 4); FPI + vehicle (N = 4); FPI + CAP (N = 4). The symbol * indicates P < 0.05.

Our previous work has identified numerous changes in cytokines and chemokines in the CNS following an FPI [32]. Because CAP reduced the number of activated splenocytes following FPI, and activated splenocytes are a potential source of cytokines and chemokines, we sought to determine if CLIP antagonism using CAP modifies the cytokine milieu in the CNS. In order to address this, we screened for multiple cytokines, using a Multiplex cytokine assay system (MCYTOMAG-70 K-PMX). The results showed that CAP treatment significantly reduced IL15, IL13, IL10 and monocyte chemoattractant protein (MCP1), also known as CCL2 (Figure 4).

**Figure 5:**
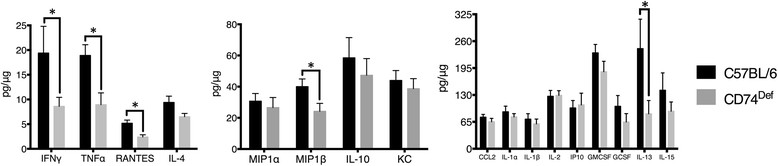
**CD74** ^**Def**^ **mice are resistant to FPI-induced increases in pro-inflammatory cytokines in the brain following TBI.** Multiplex analysis of cytokine expression in tissue from the ipsilateral cortex, comparing the cytokine response to FPI in wild type C57BL/6 mice to CD74^Def^ mice. The results demonstrate a significant decrease in the levels of TNFα, IFNγ, RANTES, MIP1β, and IL-13 in tissue from the ipsilateral cortex of CD74^Def^ mice compared to C57BL/6 mice, measured at 24 hours post FPI. The reduction in this group of cytokines in the CD74^Def^ mice, implicate CD74 in their production following FPI. For these experiments we used five C57BL/6 and six CD74^Def^. The symbol * indicates P < 0.05.

Because the absence of invariant chain in the CD74^def^ mice resulted in no splenocyte activation after FPI, we similarly sought to examine the cytokine and chemokine expression in the brains of these KO mice after FPI. We observed significantly less TBI-induced expression of IFNγ, TNFα, regulated on activation normal T cell expressed and secreted (RANTES; AKA CCL5), macrophage inflammatory protein 1β (MIP1β; AKA CCL4) and IL-13, in the CNS of CD74^def^ mice when compared to C57BL/6 mice (which are the genetic background of these mice) (Figure 5).

### Invariant chain deficiency or CLIP antagonism confers neuroprotection from FPI

**Figure 6:**
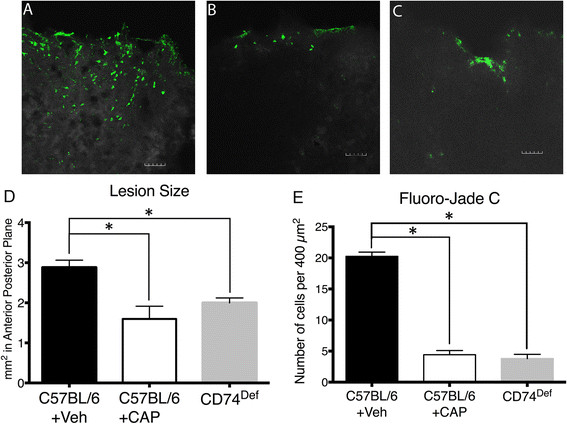
**CAP treatment after FPI reduces neurodegeneration and lesion size. (A-C)** FluoroJade C labeling at 3 days after FPI. In vehicle treated mice **(A)**, significantly more degenerating neurons are observed compared to mice treated with CAP after FPI **(B)** and CD74^def^ mice **(C)**. **(D)** Analysis of the size of the lesion at 3 days after FPI revealed that CAP treatment and CD74^Def^mice had a significantly smaller lesion in the anterior/posterior plane, compared to sham mice. **(E)** Quantitative analysis of the number of FluorJade C-labeled cells showed significantly less (P < .01 for both) labeled-cells in the CAP treated and CD74^Def^ mice. For this analysis, we used 6 shams, 5 CAP and 5 CD74^Def^mice. Scale bars in **A-C** = 50 μm. The symbol * indicates P < 0.01.

To determine the contribution of CD74 to FPI-induced neurodegeneration, we examined FluoroJade C labeling at 3 days after FPI in WT and CD74^Def^ mice. The 3 day time point was selected because we previously demonstrated that this was the peak of neurodegeneration following FPI [26]. The results demonstrate that relative to vehicle treatment (Figure 6A), treatment with CAP significantly reduced the number of FluoroJade C-labeled cells in the ipsilateral cortex (Figure 6B). Similarly, mice deficient for CD74 (Figure 6C) had decreased FluoroJade C-labeling compared to vehicle treatment (Figure 6A). In addition, Analysis of the overall lesion size revealed that both, CAP treated and CD74^def^ mice had a significantly smaller lesion size compared to WT mice (Figure 6D). Quantitation of Fluorojade-staining in the brain following CAP administration and in CD74^def^ mice, demonstrated a significant reduction in degenerating cells (Figure 6E).

## Discussion

This study is the first to demonstrate a putative role for invariant chain CD74 in CNS damage following brain injury. The data support targeting the transition between innate and adaptive immunity in the periphery as a potential therapeutic approach for TBI. Alterations to peripheral immune cell populations include an increase in cellularity in the spleen, comprised of increased B cells, CD3+ T cells, CD4+ T cells, CD8+ T cells, Tregs and γδ T cells. Further, we found that TBI induces a decrease in cell surface CLIP on B220+ B cells, and a decrease in MHCII on activated γδ T cells, suggesting that B cells may be the dominant APC after TBI. We also determined by using CD74^Def^ mice, that these changes were invariant chain dependent.

The fact that CLIP antagonism and CD74^Def^ mice have a reduced lesion size and decreased neurodegeneration after TBI, supports the involvement of CLIP and/or CD74 in mediating damage in the CNS. Both CD74 and its products, including CLIP, are widely accepted as central players in antigen processing and presentation, and are central to the transition between innate and adaptive immunity [12]. The neuroprotection observed in this study supports the involvement of antigen processing and presentation in the exacerbation of brain injury [17],[18],[33],[34]. While it is clear that CD74 is involved in the damage to the CNS following TBI, several different functions have also been attributed to CD74. These include: survival, signal transduction through cell surface CD74, binding to pro-inflammatory macrophage inhibitory factor (MIF), along with its well-established role in antigen processing and presentation [353 day CitationID="CR38">38]. Based on the current findings, it is not possible to exclude any of these functions of CD74 in mediating neurodegeneration after TBI.

To further elucidate the putative role of CD74 in TBI, we engineered a peptide that competitively antagonizes the proteolytic product of CD74, CLIP [25]. We determined that administration of this potentially therapeutic peptide 20 minutes after TBI prevented the increase in splenic cellularity in all observed peripheral immune cell populations with the exception of activated conventional CD4+ T cells. Most importantly, administration of this peptide was neuroprotective following TBI, supporting the notion that neuronal damage involves the transition between innate and adaptive immunity.

The transition between innate and adaptive immunity can be regulated by several subsets of activated, non-conventional T cells, including Tregs and γδ T cells. While Tregs have been well described to dampen antigen-specific responses in the periphery, their role in the CNS is controversial [39]-[41]. Moalem et al. has proposed that following injury to the nervous system, Tregs may be detrimental, because the antigen specific T cell response that they limit may be protective [18],[19],[33]. Alternatively, some investigators have proposed protective effects of Tregs following cerebral insults [40]-[43]. It is pertinent to note that two distinct subsets of Tregs have been characterized, natural Tregs and inducible Tregs [44],[45]. Therefore, it is possible that one population of Tregs is beneficial and the other is detrimental. Future studies are needed to examine this possibility.

The current results also demonstrated a significant expansion of γδ T cells after TBI that could be reversed with CAP treatment. Freedman and colleagues previously demonstrated the presence of γδ T cells in the cerebral spinal fluid of patients with multiple sclerosis [46]. Additionally, it has previously been shown that these γδ T cells could attack oligodendrocytes, indicating the potential for a destructive response by γδ T cells in the CNS [46],[47]. Our data showing a reduced MHCII expression by γδ T cells after FPI argues for reduced capability for antigen presentation and processing. This reduced ability for antigen presentation and processing could prevent an effective transition from innate to adaptive immunity. Such prevention of an effective transition to adaptive immunity could undermine what would otherwise be protective auto-immunity as previously suggested [18],[33].

Similar to γδ T cells, B cells are also well described as antigen specific, capable of producing antibodies, and as exquisitely efficient professional antigen presenting cells [21],[22]. Our finding that after FPI, B cells have significantly reduced CLIP, but not significantly reduced MHCII, suggests that these cells have a higher likelihood for antigen presentation after TBI [22]. In support of this notion, a recent study showed that the antigen presentation function of B cells is critical for the pathology of experimental autoimmune encephalitis (EAE), more so than auto-antibody production [34]. Thus, some consequences of TBI might depend upon efficient antigen processing and presentation by B cells.

The effector functions of B cells and T cells involve cytokines and chemokines. These small molecule compounds serve as chemotactic signaling proteins and immune modulators. Numerous studies have implicated a variety of chemokines and cytokines in the pathogenesis of TBI [32]. The interactions that govern the effects of cytokines are poorly understood in general, and even less-well understood in the context of TBI. In CD74^Def^ mice, we observed significantly less IFNγ and TNFα compared to WT controls following FPI. Elevations of both of these cytokines have been previously reported following various types of TBI [48]-[52], but the cellular source(s) of these cytokines and their functional significance are not well-defined. While some studies demonstrate that glial cells, including microglial cells are involved in the increase [53],[54], the possibility that infiltrating immune cells, including macrophages, contribute to these increases cannot be ruled out. In support of this interpretation, we also observed decreased MIP1β (Figure 5) and RANTES, both of which are products of CD8+ T cells [55],[56], which were also reduced with CAP (Figure 1). Nevertheless, our data with CD74^Def^ mice implicate antigen processing involving CD74 as a contributor to the elevation of these cytokines and chemokines after TBI.

While CCL2 is a well-described marker of inflammation after TBI [57],[58], and CAP reduced its expression in this study, less understood is the putative roles of IL15, IL13 and IL10, that were also reduced by CAP. Interestingly, a previous study using IL15 KO mice and IL15R KO mice in a facial axotomy model, found reduced T cell infiltration and increased MHCII + microglial cells [59]. Consistent with the potential involvement of macrophages/microglial cells, IL13 has been previously shown to be involved in the elimination of activated microglial cells subsequent to an injury [60]. In our study, administration of CAP reduced the lesion size, which could reflect a reduction in the number of activated microglial cells responding to the injury, thereby decreasing the overall level of IL15 and IL-13. With regard to the CAP-dependent decrease in IL-10, previous studies have indicated that elevated IL-10 corresponds with a poor outcome for patients [61],[62]. Therefore, the reduction in IL-10 observed following CAP treatment may be indicative of the decreased lesion size and thus an improved outcome.

## Conclusion

The synthesis of CAP was based on the rationale that antagonism of CLIP would selectively target the transition between innate and adaptive immunity. While our results are consistent with this hypothesis, the consequences of CAP therapy resulting in neuroprotection require further examination to fully elucidate the precise mechanisms involved. Nonetheless, the results from the current study provide a foundation for targeting the switch between innate and adaptive immunity for the treatment of TBI. Moreover, our specific characterization of peripheral alterations to distinct subsets of immune cells may provide biomarkers for diagnosing and characterizing the degree of TBI severity, progression and/or recovery.

## Authors’ contributions

RPT helped design experiments, performed experiments, analyzed data, and helped write the manuscript. SM helped design and perform experiments. JMK performed cytokine multiplex experiments. SKR, SKH, and HLM performed experiments. MKNR conceived the study, designed experiments, analyzed data, and helped write the manuscript. LAS helped conceive the study, designed experiments, analyzed data, and helped write the manuscript. All authors have read and approved the final manuscript.

## Authors’ information

RPT is a postdoctoral fellow, Department of Surgery, Texas A&M University College of Medicine. SM is a research scientist, Department of Surgery, Texas A&M University College of Medicine. JMK is a research assistant, Department of Surgery, Central Texas Veterans Health Care System, Texas A&M University College of Medicine. SKR is an undergraduate student, Department of Anthropology, University of Texas, Austin. SKH is a research assistant, Department of Surgery, Baylor Scott and White Health. HLM is a research assistant, Department of Surgery, Texas A&M University College of Medicine. MKNR is a Professor, Department of Surgery, Texas A&M University College of Medicine, and Baylor Scott and White Health. LAS is an Assistant Professor, Department of Surgery, Central Texas Veterans Health Care System, Texas A&M University College of Medicine.

## Additional files

## Electronic supplementary material

Additional file 1: Figure S1.: Flow cytometric-gating strategy. Representative 5% contour plots of flow cytometric data to show gating strategy. (TIFF 1 MB)

Additional file 2: Figure S2.: The impact of CAP on the cell surface levels of CLIP and MHCII on B and γδ T cells. **(A)** The mean fluorescence intensity (MFI) of CLIP on the surface of B cells in the spleens of C57BL/6 mice 24 hours following sham surgery or FPI. **(B)** The number of CLIP^+^ B cells (B220^+^CLIP^+^). **(C)** The MFI of MHCII on the surface of B cells in the spleens of C57BL/6 mice 24 hours following sham surgery or FPI. **(D)** The MFI of CLIP on the surface of γδ T cells in the spleens of C57BL/6 mice 24 hours following sham surgery or FPI. (E) The MFI of MHCII on the surface of γδ T cells in the spleens of C57BL/6 mice 24 hours following sham surgery or FPI. Sham + CAP (N = 5); FPI + vehicle (N = 6). (TIFF 595 KB)

Below are the links to the authors’ original submitted files for images.

Authors’ original file for figure 1

Authors’ original file for figure 2

Authors’ original file for figure 3

Authors’ original file for figure 4

Authors’ original file for figure 5

Authors’ original file for figure 6
