# Supplementary figures and images for: Traumatic brain injury causes selective, CD74-dependent peripheral lymphocyte activation that exacerbates neurodegeneration
Source: Acta Neuropathol Commun. 2014 Oct 20;2:143. doi: 10.1186/s40478-014-0143-5 (PMC4203873; doi:10.1186/s40478-014-0143-5)

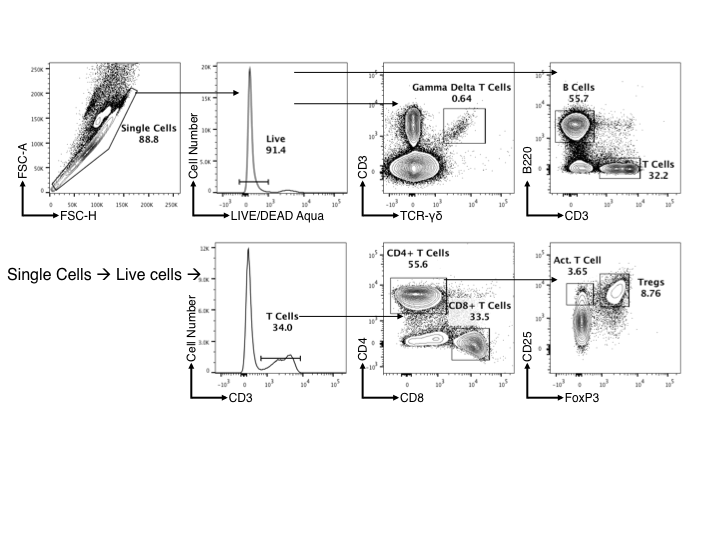

Supplement: Supplementary file 1 — Additional file 1: Figure S1.: Flow cytometric-gating strategy. Representative 5% contour plots of flow cytometric data to show gating strategy. (TIFF 1 MB) [file 40478_2014_9143_MOESM1_ESM.tiff]

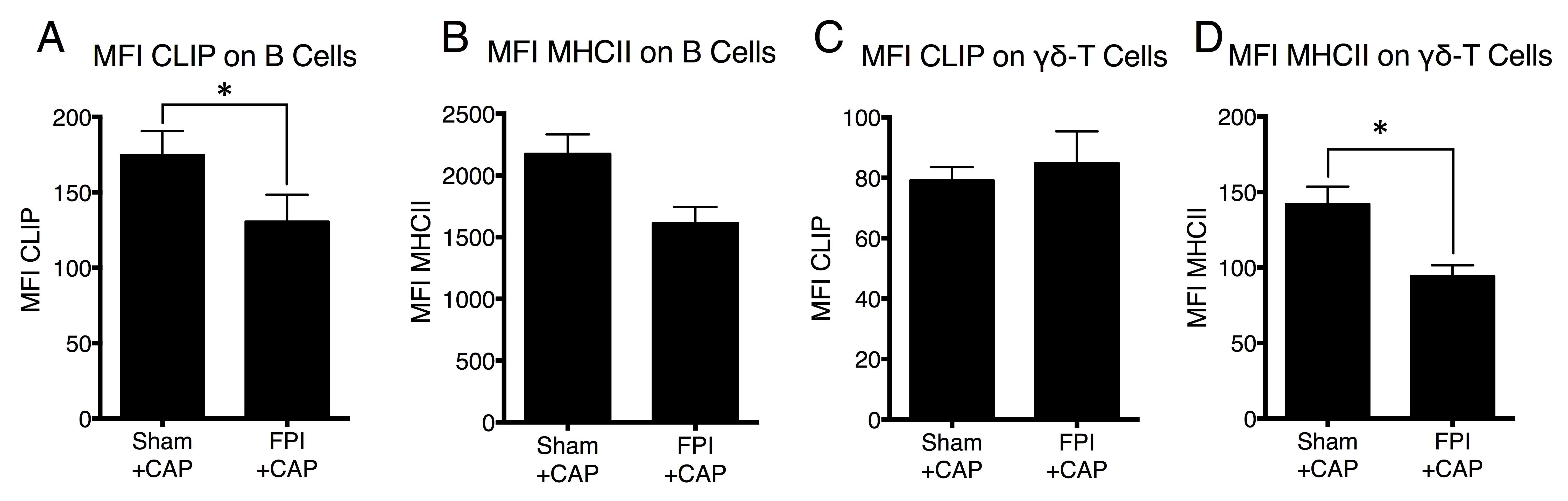

Supplement: Supplementary file 2 — Additional file 2: Figure S2.: The impact of CAP on the cell surface levels of CLIP and MHCII on B and γδ T cells. (A) The mean fluorescence intensity (MFI) of CLIP on the surface of B cells in the spleens of C57BL/6 mice 24 hours following sham surgery or FPI. (B) The number of CLIP+ B cells (B220+CLIP+). (C) The MFI of MHCII on the surface of B cells in the spleens of C57BL/6 mice 24 hours following sham surgery or FPI. (D) The MFI of CLIP on the surface of γδ T cells in the spleens of C57BL/6 mice 24 hours following sham surgery or FPI. (E) The MFI of MHCII on the surface of γδ T cells in the spleens of C57BL/6 mice 24 hours following sham surgery or FPI. Sham + CAP (N = 5); FPI + vehicle (N = 6). (TIFF 595 KB) [file 40478_2014_9143_MOESM2_ESM.tiff]

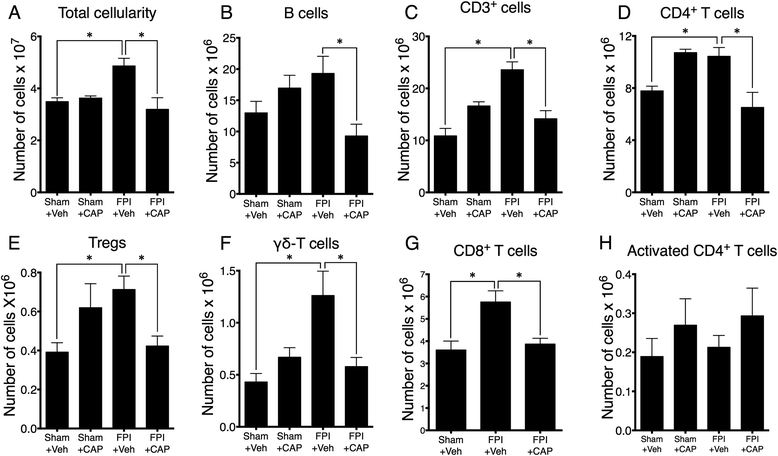

Supplement: Supplementary file 3 — Authors’ original file for figure 1 [file 40478_2014_9143_MOESM3_ESM.gif]

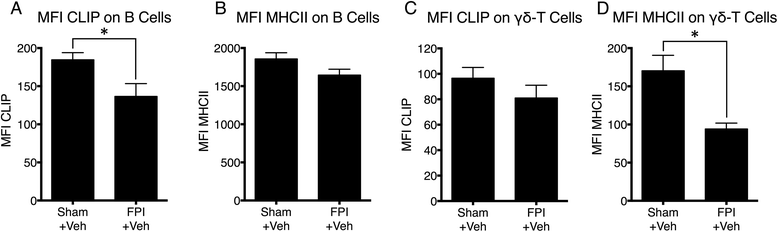

Supplement: Supplementary file 4 — Authors’ original file for figure 2 [file 40478_2014_9143_MOESM4_ESM.gif]

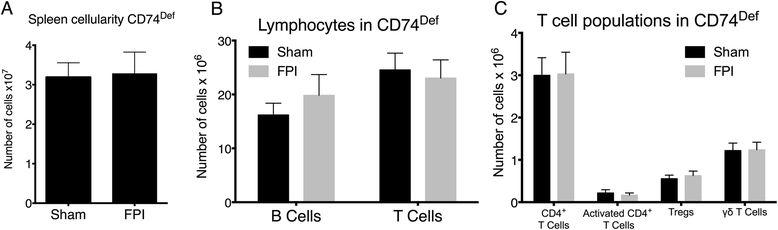

Supplement: Supplementary file 5 — Authors’ original file for figure 3 [file 40478_2014_9143_MOESM5_ESM.gif]

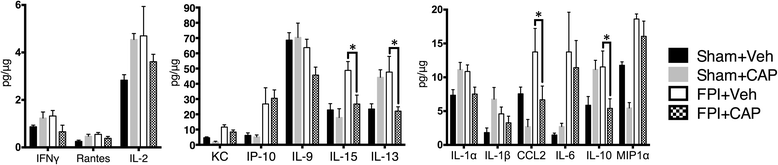

Supplement: Supplementary file 6 — Authors’ original file for figure 4 [file 40478_2014_9143_MOESM6_ESM.gif]

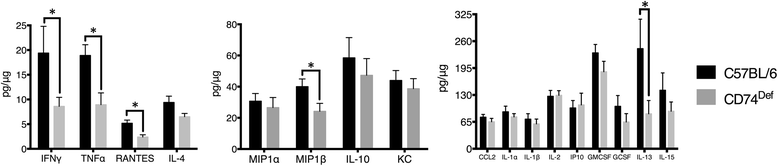

Supplement: Supplementary file 7 — Authors’ original file for figure 5 [file 40478_2014_9143_MOESM7_ESM.gif]

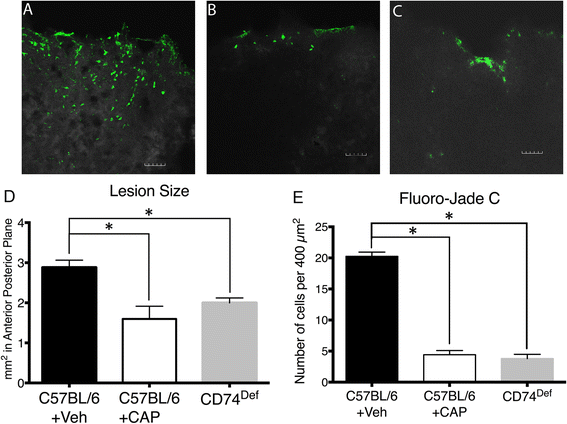

Supplement: Supplementary file 8 — Authors’ original file for figure 6 [file 40478_2014_9143_MOESM8_ESM.gif]
